# Evaluating the efficacy and specificity of PVPP for phenolic compound removal from natural organic matter

**DOI:** 10.1371/journal.pone.0355392

**Published:** 2026-08-10

**Authors:** Alexis E. Slentz, Robert G. M. Spencer, Rachel M. Wilson, Malak M. Tfaily, Caitlin Petro, Joel Kostka, Jeffrey P. Chanton

**Affiliations:** 1 Department of Earth, Ocean, and Atmospheric Science, Florida State University, Tallahassee, Florida, United States of America; 2 Geochemistry Group, National High Magnetic Field Laboratory, Tallahassee, Florida, United States of America; 3 Department of Environmental Science, University of Arizona, Tucson, Arizona, United States of America; 4 School of Biological Sciences and School of Earth and Atmospheric Sciences, Center for Microbial Dynamics and Infection, Georgia Institute of Technology, Atlanta, Georgia, United States of America; National Research and Innovation Agency, INDONESIA

## Abstract

Experimental tests of the enzyme latch hypothesis frequently rely on the removal of phenolic compounds using the phenol binding agent polyvinylpyrrolidone (PVP), yet the extent to which PVP-based approaches alter the composition of the broader dissolved organic matter (DOM) pool remains poorly constrained. Here, we evaluated the selectivity of solid phase polyvinylpolypyrrolidone (PVPP) using Suwannee River Fulvic Acid (SRFA) as a highly characterized representative DOM standard and, for the first time, combined bulk measurements of dissolved organic carbon (DOC) and phenolic removal with Fourier transform ion cyclotron resonance mass spectrometry (FT-ICR MS) characterization of residual DOM before and after PVPP treatment. Phenolic binding efficiency increased systematically with PVPP amount and column conditioning, with a rehydrated 1.5 g PVPP-packed column reducing soluble phenolic concentrations by 91%, DOC by 61%, and C-normalized phenolic content from 21% to 5% of the DOM pool. FT-ICR MS analysis revealed that PVPP preferentially removed polyphenolic and condensed aromatic molecular formulae, while leaving the residual DOM enriched in highly unsaturated and aliphatic constituents. These compositional shifts were accompanied by decreases in aromaticity, double-bond equivalents, nominal oxidation state of carbon, and average molecular mass, indicating selective sequestration of aromatic, higher molecular weight DOM. Our results demonstrate that PVPP acts as a strongly selective, but not perfectly specific, adsorbent for phenolic-rich DOM and substantially alters the composition of the remaining DOM pool. This work provides a molecular-level framework for interpreting PVP/PVPP-based phenolic manipulation experiments and establishes best practice guidance for their application in peatland and aquatic biogeochemical research.

## Introduction

Phenolic compounds have been proposed as potent regulators of microbial respiration in peatlands due to their apparent disruption of extracellular enzyme activity under anoxic conditions, a framework commonly referred to as the “enzyme latch” hypothesis [1,2]. While the enzyme latch is generally accepted, there are still aspects of this phenomenon that remain unresolved. Importantly, the extent to which phenolic compounds inhibit organic matter decomposition is unclear, and studies evaluating the enzyme latch mechanism have yielded mixed results [2–6]. For example, while Freeman et al. [2] demonstrated that reduced phenolic concentrations in peat porewater can increase hydrolytic enzyme activity by 20–47%, Urbanová and Hájek [3] show that increasing concentrations of phenolic-rich organic matter to peat incubations has minimal effect on enzyme activity or respiration rate compared to a control, and McGivern et al., [5] found that polyphenol metabolism could contribute to, rather than inhibit, peatland soil respiration. However, experimental inconsistencies may reflect differences in approaches used to isolate phenolic effects, rather than fundamental disagreements about phenolic inhibition of peat decomposition.

A number of investigations on the controls on peat decomposition by phenolic compounds rely on their removal using polyvinylpyrrolidone (PVP) or its insoluble form, polyvinylpolypyrrolidone (PVPP) [2,4]. PVP-based approaches have been shown to effectively bind and remove dissolved phenolics from diverse aqueous solutions [7–10], leading to their adoption as a standard tool for isolating phenolic effects in peatland biogeochemical studies. However, despite their widespread use, the selectivity of PVP-based approaches with respect to the broader dissolved organic matter (DOM) pool remains poorly constrained. Critically, it remains unclear whether PVP removes phenolic compounds exclusively or binds to DOM indiscriminately, altering molecular composition in unintended ways and potentially confounding interpretations of phenolic manipulation experiments. To our knowledge, no previous study has combined bulk measurements of DOC and phenolic removal with molecular-level characterization of residual DOM before and after PVPP treatment to evaluate the selectivity of this widely used phenolic manipulation approach. As a result, the compositional consequences of PVPP treatment for the remaining DOM pool remain poorly understood, limiting interpretation of PVP/PVPP-based manipulation experiments and efforts to reconcile conflicting findings regarding phenolic controls on peatland decomposition.

The objective of this study was to critically assess a commonly used DOM manipulation approach employed in peatland carbon cycling studies: the removal of dissolved phenolic compounds using PVP. To this end, we evaluated a simple and efficient means for the operational removal of phenolic compounds from natural DOM by employing water-insoluble polyvinylpolypyrrolidone (PVPP) as a column packing material. This investigation utilized a well-known, readily available, and highly characterized environmental standard reference material, the International Humic Substances Society (IHSS) Suwannee River Fulvic Acid (SRFA) [11–14]. Sequential aliquots of the SRFA solution were treated with varying amounts of PVPP under different hydration regimes to determine the best-practice phenol removal technique, which was evaluated using changes in concentrations of dissolved organic carbon (DOC; the commonly quantified fraction of DOM) and phenolic compounds following each treatment. The influence of PVPP on overall DOM molecular composition was determined using Fourier Transform ion cyclotron resonance mass spectrometry (FT-ICR MS), enabling molecular-level insights into broad compositional changes across the DOM pool and providing a unique opportunity to assess the selectivity and specificity of PVPP- information not accessible through bulk DOC or total phenolic measurements alone. Utilizing the response of a standard reference material, this study provides an empirical basis for evaluating the selectivity and interpretability of PVP-based phenolic manipulation experiments within the context of the enzyme latch hypothesis.

## Materials and methods

### Removal of phenolic compounds

**Fig 1:**
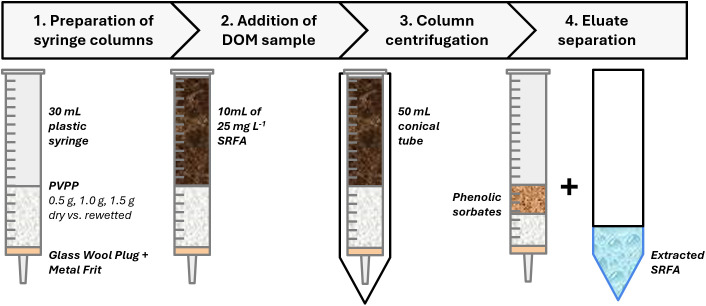
Experimental set-up for the extraction of phenolic compounds from natural organic matter samples using a PVPP-packed column.

Three different quantities of polyvinylpolypyrrolidone (PVPP; Sigma-Aldrich Chemical Co., St. Louis, MO, USA; 0.5 g, 1.0 g, and 1.5 g) and two levels of hydration (1.5 g dry, 1.5 g rehydrated; triple-rinsed with Milli-Q water (18.2 MΩ) prior to use) were used to hone the extraction procedure. Following a modified version of the protocol outlined by Ranatunge et al. [15], the PVPP was pre-weighed and packed into a pre-cleaned (10% HPLC-grade HCl, > 24 h) 30 mL syringe barrel following the removal of the plunger and the addition of a metal frit and a small layer of quartz wool (Fig 1). A 10 mL aliquot of a 25 mg L^-1^ (13.94 mg C L^-1^) stock solution of Suwannee River Fulvic Acid (SRFA; standard number 2S101F; International Humic Substance Society) was layered on top of the PVPP, and the column was placed into a 50 mL conical centrifuge tube. Each SRFA-loaded column was centrifuged for 10 minutes at 6000 rpm, and the resulting supernatant was collected and filtered to 0.7 µm (Whatman GF/F, pre-combusted at 450°C > 4 h; Cytiva, Malrborough, MA, USA). The first aliquot was discarded for all experiments because the initial application of sample to the column resulted in substantial adsorption to the packing material, leading to non-representative recovery [15]. The process was repeated five more times to determine extraction efficiency with continued use, each aliquot after the first being collected and filtered into separate vessels. Specifically, each column extraction was conducted in triplicate (i.e., three columns prepared for each PVPP amount/condition), and six total consecutive sample aliquots were run through each column without further modification to the PVPP between each addition. All supernatant aliquots and replicates, in addition to the crude SRFA stock solution, were analyzed for total soluble phenolics as described below. Replicates for a given aliquot were combined into a larger singular sample prior to DOC measurements and molecular-level characterization due to volume constraints.

### Dissolved organic carbon analysis

DOC concentrations of filtered (0.7 µm) and acidified (HPLC-grade 10 M HCl, pH = 2) SRFA samples were measured on a Shimadzu TOC-L CPH high-temperature catalytic oxidation total organic carbon analyzer (Shimadzu Corp., Kyoto, Japan). Following established methodology each sample was sparged with ultrapure air at a rate of 80 mL min^-1^ for 8 minutes, after which the non-purgeable organic carbon (i.e., DOC) was averaged over three to seven replicate injections (50 µL) with a coefficient of variance <2% [16,17].

### Soluble phenolic analysis

Concentrations of soluble phenolics were determined in accordance with the Folin-Ciocalteu (FC) colorimetric assay as described by Bancuta et al. [18]. A 0.1 mL aliquot of each filtered (0.7 µm) sample was added to sterile micro centrifuge tubes, into which 0.5 mL of 0.27 M FC reagent (MP Biomedicals, Solon, OH, USA) was mixed and allowed to sit for five minutes. Then, 0.5 mL of 93.6 g L^-1^ Na_2_CO_3_ (VWR Chemicals, LLC, Solon, OH, USA) was added and mixed. After two hours, solution absorbance at 765 nm was measured using a BioTech Synergy HTX Multi-Mode Reader UV/Vis absorbance spectrophotometer (Agilent, Santa Clara, CA, USA). Phenolic content was quantified using a seven-point gallic acid calibration curve (3.0–60.0 mg L^-1^) as described previously [4].

### Fourier transform ion cyclotron resonance mass spectrometry

DOM was isolated in preparation for FT-ICR MS analysis following established protocols [19] using solid-phase extraction (SPE) onto individual 100 mg bed, 3 mL volume Bond-Elut PPL cartridges (Agilent Technologies Inc., Santa Clara, CA). Cartridges were rinsed and then saturated with HPLC grade methanol (Sigma-Aldrich Chemical Co., St. Louis, MO, USA) overnight and then rinsed with ultrapure water, methanol, and pH 2 ultrapure water. Assuming an extraction efficiency of at least 55% [20], acidified (pH 2) sample aliquots equivalent to a targeted 40 µg C were extracted onto the prepared cartridges and eluted with HPLC grade methanol into pre-cleaned (10% HCl v/v, 48 h) and combusted (550°C, > 5 h) 2 mL amber glass vials. Methanolic extracts were stored at −20°C until analysis.

DOM extracts were analyzed without further modification on a custom-built hybrid linear ion trap 9.4 T FT-ICR mass spectrometer at the National High Magnetic Field Laboratory (Tallahassee, FL) [21]. Negatively charged ions were produced using electrospray ionization at a flow rate of 500 nL min^-1^ and emitter voltage of −3.2 kV, collected over 100 coadded scans using Predator data station [22]. Mass spectra were phase-corrected [23] and experimentally measured masses were converted to the Kendrick mass scale [24] for rapid identification of homologous series for each heteroatom class (i.e., species with the same C_c_H_h_N_n_O_o_S_s_ content, different only by degree of alkylation) [25]. Peaks were internally calibrated using 10–15 highly abundant O-containing homologous series spanning the entire mass range (200–1000 Da) using a “walking” calibration [26]. Mass spectral peaks (>6σ root-mean square baseline noise at m/z 400) were exported to peak lists, and molecular formula assignments and data visualization were performed with PetroOrg software [27–29].

Molecular formulae were assigned to ions constrained by C_4-75_H_4-150_O_1-30_N_0-4_S_0-2_. For all mass spectral data presented herein, 8,753−10,436 peaks were assigned elemental compositions (error ± 0.3 ppm). The modified aromaticity index (AI_mod_; a robust proxy for aromaticity) and nominal oxidation state of carbon (NOSC) were calculated for each formula according to Koch and Dittmar [30,31] and Boye et al. [32], respectively. Stoichiometric ratios (H/C, O/C, N/C and S/C) were calculated, and formulae were grouped based on their heteroatom content (CHO, CHON, CHOS, and CHONS). Molecular formulae were also grouped into operational compound classes based on elemental ratios (H/C and O/C) and AI_mod_: condensed aromatics (CA; AI_mod_ > 0.67), polyphenolics (PPh; 0.50 < AI_mod_ < 0.67), highly unsaturated low-O/C (HU_low o/c_; AI_mod_ < 0.50, H/C < 1.5, O/C < 0.5), highly unsaturated high-O/C (HU_high o/c_; AI_mod_ < 0.50, H/C < 1.5, O/C > 0.5), and aliphatic (Ali; H/C > 1.5) [33]. The relative abundance (RA) of each assigned formula was determined by expressing its peak intensity as a proportion of the total intensity of all assigned peaks in the sample, and the % RA of each heteroatom and operational compound class was calculated by summing the RA of all peaks belonging to the respective class.

## Results and discussion

### Methodological consideration: column conditioning and contaminant removal

**Fig 2:**
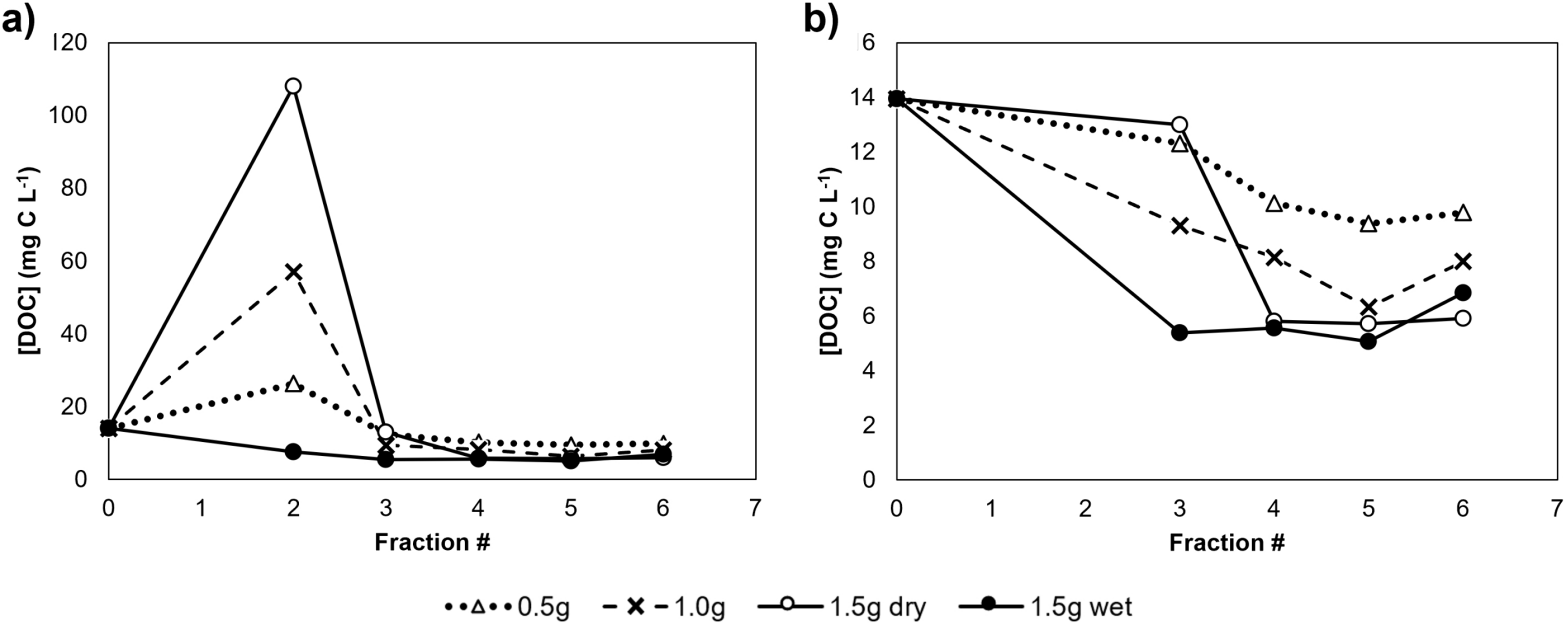
Average DOC concentrations of supernatant aliquots following repeat usage of PVPP extraction columns. Panel a) includes all collected sequential fractions, while panel b) excludes the anomalous F2. In both panels the aliquot number (x-axis) is the n^th^ time the column was employed, and the y-axis is the concentration of residual DOC which passed through the column following the addition of a 10 mL aliquot of a 13.94 mg C L^-1^ stock solution of SRFA. Open triangles with a dotted line represent 0.5 g PVPP columns, “X” points with a dashed line represent 1.0 g PVPP columns, open circles with a solid line represent 1.5 g dry PVPP columns, and filled circles with a solid line represents 1.5 g rewetted PVPP columns.

A significant spike in DOC concentrations was observed in the second sequential fractions (F2) of column supernatants, specifically from the “dry” PVPP-packed columns (Fig 2a). DOC concentrations increased by 12.33 mg C L^-1^ for 0.5 g PVPP columns, 42.97 mg C L^-1^ for 1.0 g PVPP columns, and 94.06 mg C L^-1^ for 1.5 g PVPP columns (Table 1 and Fig 2). The increase in DOC concentration significantly (*p* = 0.01) and proportionally corresponded with the 0.5 g increase in the amount of PVPP used in each column (+19.02 mg C g PVPP^-1^), suggesting that some amount of soluble PVP or another soluble contaminant was present in the PVPP used as column packing material. This increase in DOC was not observed in the rehydrated 1.5 g PVPP columns (Table 1 and Fig 2a), indicating that adequate rinsing of the PVPP with Milli-Q water is necessary to remove any soluble PVP or other contaminants that may be present in PVPP resins prior to use. Based on these findings, F2 was discarded in addition to F1, regardless of each column’s initial hydration state to ensure aliquots were free of contaminants prior to subsequent analysis (Fig 2b).

**Table 1:** Average experimental dissolved organic carbon (DOC) and total phenolic content (Ph). Concentrations ([DOC] and [Ph]) are shown for the initial SRFA stock solution and the n^th^ PVPP-treated supernatant aliquots of each column extraction experiment, as well as amounts lost relative to the initial concentration (Δ[DOC] and Δ[Ph]) and associated percent change.

Column Experiment	Fraction #	[DOC] (mg C L^-1^)	Δ[DOC] (mg C L^-1^)	DOC % Change	[Ph] (mg Ph L^-1^)	Δ[Ph] (mg Ph L^-1^)	Ph % Change
	**Initial**	13.94			2.964		
**0.5 g, dry**	**2**	26.27	12.33	88	2.20	−0.76	−26
	**3**	12.31	−1.63	−12	1.44	−1.53	−51
	**4**	10.13	−3.81	−27	1.20	−1.77	−60
	**5**	9.38	−4.56	−33	1.20	−1.77	−60
	**6**	9.78	−4.16	−30	1.16	−1.81	−61
**1.0 g, dry**	**2**	56.91	42.97	308	1.64	−1.33	−45
	**3**	9.31	−4.63	−33	0.96	−2.01	−68
	**4**	8.14	−5.80	−42	0.59	−2.37	−80
	**5**	6.32	−7.62	−55	0.67	−2.29	−77
	**6**	7.99	−5.95	−43	0.80	−2.17	−73
**1.5 g, dry**	**2**	108.00	94.06	675	2.32	−0.64	−22
	**3**	13.00	−0.94	−7	0.71	−2.25	−76
	**4**	5.80	−8.15	−58	0.39	−2.57	−87
	**5**	5.72	−8.22	−59	0.43	−2.53	−85
	**6**	5.90	−8.04	−58	0.39	−2.57	−87
**1.5 g, wet**	**2**	7.46	−6.48	−46	0.51	−2.45	−83
	**3**	5.38	−8.56	−61	0.27	−2.69	−91
	**4**	5.55	−8.39	−60	0.35	−2.61	−88
	**5**	5.06	−8.88	−64	0.35	−2.61	−88
	**6**	6.84	−7.10	−51	0.27	−2.69	−91

### Bulk DOC removal by PVPP

The initial DOC concentration of the 25 mg SRFA L^-1^ stock solution was measured at 13.94 mg C L^-1^; this aligns well with the estimated elemental composition of the SRFA standard (i.e., 52.34% C as reported by IHSS). Increasing amounts of PVPP packed into the columns led to lower DOC concentrations following treatment of the SRFA solution. Columns packed with 0.5 g PVPP exhibited the lowest change in DOC concentration, removing a maximum of only 33% of the initial DOC concentration (−4.56 mg C L^-1^; Table 1). The use of 1.0g PVPP led to a greater removal of up to 55% of the initial DOC (−7.62 mg C L^-1^; Table 1), and the use of 1.5 g PVPP led to the greatest DOC removal; up to 59% (−8.22 mg C L^-1^, Table 1). This result was expected, as adsorption percentage increases with adsorbent amount [34,35] and has been demonstrated in previous similar studies [4,15,34,36].

Additionally, the amount of DOC removed by each column generally increased with continued use, with maximum binding efficiency being achieved by the fifth aliquot addition (F5; Table 1 and Fig 2). This increase in column efficiency with continued use was attributed to the increase in hydration level of the PVPP, which has been shown to increase binding efficiency of PVPP via increased surface area of contact between the binding agent and the sample, thus increasing available binding sites [37,38] as well as by facilitating hydrogen bonding between phenolic hydroxyl groups and PVPP carbonyl groups in addition to other intramolecular interactions [34,39–42]. In agreement with this interpretation of results, DOC removal efficiency of columns packed with 1.5 g of originally dry PVPP converged with that of columns packed with 1.5 g of rehydrated PVPP after repeated use and consequent hydration (Table 1 and Fig 2). In addition, DOC binding efficiency began to decrease by the sixth iteration of column use for all columns except for those packed with 1.5 g of originally dry PVPP (Table 1 and Fig 2). This further demonstrates that binding capacity is relative to the amount of binding agent used (i.e., the number of binding sites available). This phenomenon was also observed for the rehydrated 1.5 g PVPP columns; while adequate hydration increases effective function of PVPP as a binding agent [18,39], the observed decreased DOC removal in later aliquots may reflect rapid saturation of available binding sites due to initially high binding affinity [43].

### Phenolic removal by PVPP

**Fig 3:**
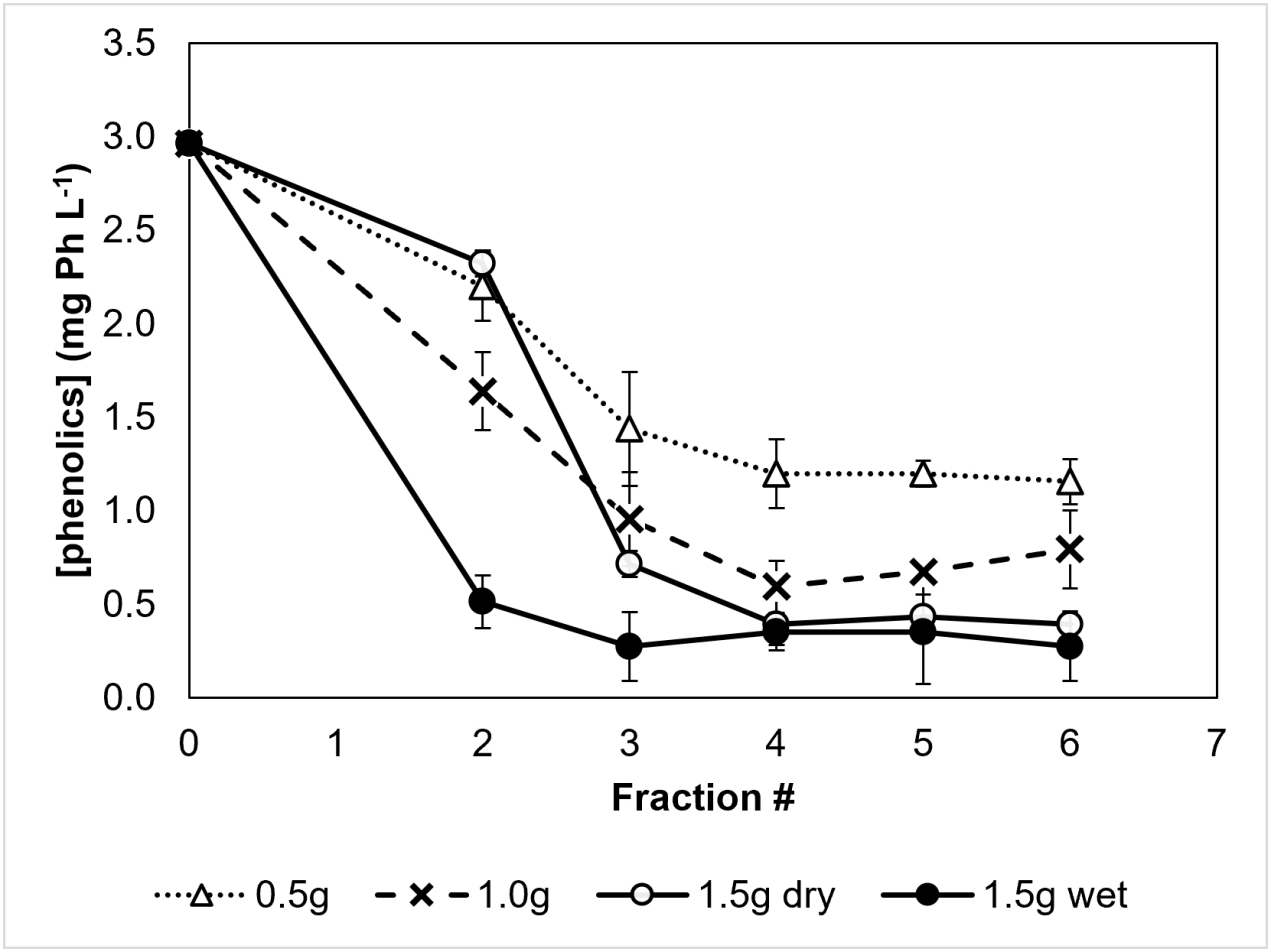
Average total phenolics concentrations with respect to gallic acid of supernatant aliquots following repeat usage of PVPP extraction columns. The aliquot number (x-axis) is the n^th^ time the column was employed, and the y-axis is the concentration of residual phenolic compounds which passed through the column following the addition of a 10 mL aliquot of a 2.96 mg Ph L^-1^ stock solution of SRFA at the head of the column. Open triangles with a dotted line represent 0.5 g PVPP columns, “X” points with a dashed line represent 1.0 g PVPP columns, open circles with a solid line represent 1.5 g dry PVPP columns, and filled circles with a solid line represents 1.5 g rewetted PVPP columns. Note that F2 is retained in this figure (unlike Fig 2b) because phenolic removal proceeded normally in F2 despite DOC contamination, demonstrating that the leached material in F2 is non-phenolic in nature.

Like DOC removal trends, phenolic binding capacity was a function of the amount of PVPP used in the columns. The initial phenolic compound concentration in the SRFA stock solution was 2.96 mg Ph L^-1^ (i.e., 21%; Table 2), corresponding with previous reports of the compound class distribution for this standard (22% aromatic via ^13^CNMR; 23.1%RA polyphenolic via FT-ICR MS) [13]. Columns packed with 0.5 g PVPP removed a maximum of 1.8 mg Ph L^-1^ (−61%; Table 1 and Fig 3), while 1.0 g PVPP reduced phenolic concentrations by up to 2.4 mg Ph L^-1^ (−80%; Table 1 and Fig 3), and 1.5 g PVPP led to a decrease by up to 2.6 mg Ph L^-1^ (−87%; Table 1 and Fig 3). Also analogous to DOC removal, the highest phenolic binding efficiency was observed in columns packed with 1.5 g of rehydrated PVPP (2.7 mg Ph L^-1^, −91%; Table 1 and Fig 3), though binding efficiency of all initially dry columns increased with continued use. By F4, phenol binding efficiency of columns packed with 1.5 g of dry PVPP converged with that of the columns packed with 1.5 g of rehydrated PVPP, further strengthening the hypothesis that PVPP hydration increases binding capacity (Fig 3) [39]. Additionally, although DOC concentrations spiked in F2 due to contaminant leaching (Fig 2), phenolic removal efficiency remained consistent across all aliquots, including F2 (Fig 3), illustrating that the leached material is non-phenolic organic carbon. This demonstrates that PVPP’s selectivity for phenolic compounds is maintained even in the presence of manufacturing-derived contaminants. Consistent across all columns, supernatant phenol concentrations remained relatively stable across F4, F5, and F6, suggesting that maximum binding efficiency for each column was reached, but the maximum binding capacity of the PVPP was not exceeded [18].

**Table 2:** Results for best practice PVPP column setup.

	Initial	Final	Δ	% change
**[Ph] (mg Ph L**^**-1**^)	2.96	0.27	−2.69	−91
**[DOC] (mg C L**^**-1**^)	13.94	5.38	−8.56	−61
**C-normalized phenolics (mg Ph/mg C)**	0.21	0.05	−0.16	−76

Changes in total phenolic content ([Ph]), DOC concentrations ([DOC]), and C-normalized phenolics associated with the third supernatant fraction (F3) of the 1.5 g rehydrated PVPP-packed columns.

To evaluate how PVPP treatment alters the relative contribution of phenolics to the remaining DOM pool following extraction, we examined changes in C-normalized phenolic content. Considering the combined DOC and total phenolic removal patterns across all column configurations, the 1.5 g rehydrated PVPP treatment most effectively minimized residual phenolic content under the conditions evaluated, reducing the C-normalized phenolic content from the initial 21% to 5% of the total DOM pool (Table 2). In this configuration, two initial 10 mL aliquots were passed through and discarded prior to supernatant collection (i.e., F3; Figs 2 and 3) to mitigate potential carryover or dilution effects. Results of this experimentally determined “best” practical use of PVPP illustrates substantial depletion of phenolic carbon relative to the bulk DOM pool and confirms that PVPP provides an efficient and effective means of depleting phenolic compounds from DOM.

Despite achieving >90% removal of soluble phenolics, 61% DOC loss, and a reduction in C-normalized phenolic content from 21% to 5% (Table 2), questions remain regarding the specificity of PVPP as a binding agent. Bulk measurements indicate preferential removal of phenolic compounds relative to bulk DOC; however, the magnitude of DOC loss (−61%; Table 2) substantially exceeds the fraction of carbon attributed to phenolic moieties in the untreated SRFA (21% of total carbon). Although this discrepancy does not definitely demonstrate removal of non-phenolic compounds, it suggests that PVPP may interact with a broader suite of compounds beyond phenolic compounds alone. PVPP interacts with phenolic hydroxyl groups primarily through dipole interactions [40,44], but similar interactions may also occur with other polar or oxygen-rich moieties [45,46]. Consequently, bulk DOC and phenolic measurements alone cannot determine whether the observed DOC loss reflects exclusively phenolic removal or whether additional DOM constituents were also sorbed. To resolve this uncertainty and to identify which molecular classes were affected by PVPP treatment, we employed FT-ICR MS to compare DOM composition before and after PVPP treatment.

### Influence of PVPP on DOM molecular composition

**Fig 4:**
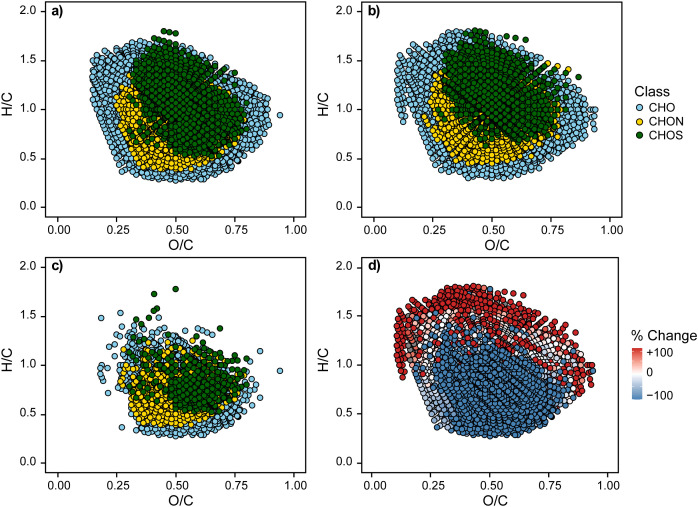
SRFA DOM molecular formulae plotted in van Krevelen space. a) Molecular composition of SRFA before treatment, b) molecular composition after PVPP treatment, c) molecular formulae unique to only SRFA before treatment, and d) the difference between pre- and post- treatment molecular composition. The color scheme in panels a-c indicates heteroatom class, where light blue points are CHO-containing formulae, yellow points are CHON-containing, and green points are CHOS-containing. The color scale in panel d illustrates the change in molecular formulae relative peak intensity as a result of PVPP treatment, ranging from a 100% decrease (blue) to a 100% increase (red).

Ultrahigh resolution mass spectrometric (i.e., FT-ICR MS) analysis of the untreated SRFA stock solution detected 10,436 unique peaks to which molecular formulae were assigned (Table 3). The average molecular mass across all peaks was 547.09 Da (Table 3). Initial stoichiometric ratios were highly comparable to previous characterization studies [13]; the weight-average H/C was 0.97 and the weight-average O/C was 0.50 (Table 3). The relative abundance (RA) of operational compound classes for SRFA were found to include 5.7% RA condensed aromatic, 29.6% RA low-O/C highly unsaturated (HU), 37.9% RA high-O/C HU, 1.1% RA aliphatic, and critically, 25.7% RA polyphenolic molecular formulae, consistent with the 21% C-normalized phenolic content observed herein and previous findings (e.g., IHSS) [13]. The modified aromaticity index (AI_mod_; a robust indicator of bulk aromaticity; [30,31]) was also consistent with Kellerman et al. [13] (0.41; Table 3). Heteroatom content was found to be less directly comparable between studies overall [13,14], though general compositional trends are consistent. Carbon, hydrogen, and oxygen-containing formulae (CHO) account for the bulk of molecular formulae present in SRFA (90.0% RA; Fig 4a), with some also including nitrogen (CHON; 7.3% RA; Fig 4a) or sulfur (CHOS; 2.7% RA; Fig 4a).

**Table 3:** Molecular formulae and FT-ICR MS parameters identified in initial and PVPP-treated SRFA samples.

FT-ICR MS Parameter	Initial	PVPP-treated
Assigned Formulae (*n*)	10436	8753
Mass (Da)	547.09	488.41
H/C	0.97	1.09
O/C	0.5	0.49
N/C	0.01	0.01
S/C	0.005	0.006
AI_mod_	0.41	0.34
DBE	14.63	11.8
NOSC	0.05	−0.1
CA (%RA)	5.7	2.2
PPh (%RA)	25.7	14.1
HU_low O/C_ (%RA)	29.6	40.8
HU_high O/C_ (%RA)	37.9	40.5
Ali (%RA)	1.1	2.6

Parameters include the number of assigned formulae, average mass, stoichiometric ratios, AI_mod_ (modified aromaticity index), DBE (double-bond equivalents), NOSC (nominal oxidation state of carbon), and the percent RA (%) of operational compound classes (CA = condensed aromatic, PPh = polyphenolic, HU_low o/c_ = low-O/C highly unsaturated, HU_high o/c_ = high-O/C highly unsaturated, Ali = aliphatic).

There were several notable changes in SRFA DOM molecular composition due to PVPP treatment (i.e., comparing the initial SRFA stock solution to the composition of DOM recovered from F3 for the 1.5 g rehydrated PVPP column). Importantly, the RA of polyphenolic and condensed aromatic compounds decreased by 11.7% RA and 3.5% RA, respectively, indicating their removal by PVPP, while aliphatic (+1.5% RA) and HU (+13.8% RA) molecular formulae were enriched in post-treatment samples (Table 3 and Fig 4d). These results provide compelling evidence that PVPP exhibited selectivity toward aromatic, phenolic-rich DOM while high-H/C DOM (aliphatic, HU molecular formulae) remained largely intact. Consequently, the weight-averaged H/C ratio increased from 0.97 in the initial SRFA solution to 1.09 after treatment (Table 3), consistent with the reported non-interaction of PVPP with aliphatic compounds (Table 3 and Fig 4) [47]. PVPP-mediated alterations to the compound classes present in SRFA DOM were accompanied by other noteworthy bulk compositional shifts, including a decrease in average molecular mass from 547.09 Da pre-treatment to 488.41 Da post-treatment, suggesting selective removal of higher-MW compounds by PVPP and supporting previous work which has shown greater binding affinity of PVPP toward highly polymerized polyphenolics [36]. The removal of aromatic moieties also resulted in an overall decrease in AI_mod_ (−0.07; Table 3), DBE (−2.83; Table 3) [48], and NOSC (−0.15; Table 3). Our findings support the results reported by Li et al. [47], which demonstrated the specific adsorption of high-MW polycyclic condensed aromatic and polyphenolic moieties by PVPP, and that DOM isolated by PVPP was more abundant in aromatic molecular formulae overall than original DOM samples (i.e., selective binding of aromatic molecular formulae relative to bulk DOM) [47].

However, the substantially greater DOC removal achieved by PVPP relative to the measured phenolic content of the original DOM pool (61% DOC removal versus 21–26% phenolic carbon) suggests that PVPP may interact with a broader range of DOM constituents than phenolic moieties alone, as previously described. In addition to polyphenolic and condensed aromatic moieties, additional DOM constituent removal was hypothesized to include more oxygen-rich molecular formulae, as many oxygen-containing functional groups are polar (e.g., hydroxyl, carboxyl, and carbonyl groups) and are thus expected to be attracted to other polar compounds, like PVPP [40,44]. PVPP displayed a very slight affinity for higher O/C compounds, leading to a minor reduction in O/C (0.50 to 0.49 post-treatment; Table 3) but negligible change in the RA of CHO-containing molecular formulae (Fig 4a-c). Heteroatom-containing compounds were overall resistant to the effects of PVPP; while the RA of CHON-containing molecular formulae was slightly reduced following PVPP treatment (−0.4% RA; Fig 4), overall N/C increased from 0.012 to 0.015 (Table 3). Similarly, both S/C and CHOS-containing molecular formulae increased slightly due to PVPP treatment of SRFA (+0.001, + 0.2% RA, respectively; Table 3 and Fig 4).

The selective removal of aromatic and polyphenolic molecular formulae observed here is consistent with a broader body of literature describing PVPP sorption behavior across environmental, brewing, and wine applications. Existing literature demonstrates that PVPP adsorbs phenolic compounds through interactions involving phenolic hydroxyl groups, with adsorption efficiency influenced by molecular structure, hydroxylation state, aromaticity, and degree of polymerization [9,35,42,44,49]. In brewing and winemaking, PVPP has long been employed to selectively remove haze-forming polyphenols and tannins [9,42], and targeted chromatographic analyses have shown preferential removal of highly hydroxylated and higher-molecular weight phenolic constituents relative to simpler phenolic compounds [44,49]. Collectively, these studies support the interpretation that PVPP acts as a selective adsorbent whose affinity is governed by chemical functionality rather than behaving as a non-specific organic matter sorbent.

Within environmental systems, Li et al. [47] provided one of the first molecular-level assessments of PVPP selectivity by characterizing the PVPP-bound fraction of Suwannee River natural organic matter using FT-ICR MS. Their results demonstrated enrichment of polyphenolic, polycyclic aromatic, oxygen-rich, and higher-molecular-weight formulae within the PVPP-retained fraction, indicating preferential sequestration of aromatic and highly unsaturated DOM constituents [47]. Our findings complement and extend this work by characterizing the residual DOM pool following PVPP treatment. While previous studies have largely focused on phenolic removal efficiency or the composition of material retained by PVPP, the effects of treatment on the broader composition of the remaining DOM pool have remained poorly constrained to this point. By combining bulk DOC and phenolic measurements with FT-ICR MS characterization before and after treatment, our results demonstrate that PVPP substantially reduces aromaticity and polyphenolic abundance while enriching the relative contribution of highly unsaturated and aliphatic molecular formulae in the residual DOM. Together, these observations provide a more complete framework for understanding how PVPP alters complex DOM mixtures and for interpreting the outcomes of phenolic manipulation experiments [1,2,4].

PVP- and PVPP-based DOM manipulation experiments have been implemented as tools for investigating phenolic controls on peatland carbon cycling and examining the efficacy of the enzyme latch hypothesis [1,2]. For example, Cory et al. [4] investigated the influence of increasing amounts of PVP added to anaerobic microcosm peat incubations on rates of CO_2_ and CH_4_ production. However, like Cory et al. [4], many of these studies have inferred reductions in inhibitory phenolic compounds from PVP or PVPP additions without directly characterizing the molecular composition of the DOM constituents removed by treatment. Instead, treatment effects have generally been evaluated using bulk measurements of phenolic abundance, extracellular enzyme activity, or carbon mineralization responses [1,2,4,50]. At the same time, studies revisiting the enzyme latch hypothesis have highlighted uncertainty about which components of complex humic and phenolic mixtures and potential other mechanisms are responsible for observed slow rates of peat OM decomposition [3,51]. In this context, our results provide molecular-level evidence that PVPP preferentially removes aromatic and polyphenolic constituents while substantially altering the composition of the residual DOM pool [47]. These findings suggest that positive responses observed in PVP- and PVPP-based experiments likely reflect the alleviation of inhibition associated with a chemically distinct subset of aromatic and polyphenolic compounds, rather than a uniform and specific reduction in total phenolic abundance. Consequently, variation in the composition and abundance of these compounds among peatlands may contribute to differences in the magnitude of responses observed across enzyme-latch investigations [1,2,4].

Interpretation of these PVPP-induced compositional shifts must be considered within the analytical window of FT-ICR MS. Despite its unparalleled resolution and mass accuracy, FT-ICR MS is inherently influenced by ionization and sample preparation biases. The use of negative mode electrospray ionization (ESI) preferentially detects polar, acidic, and unsaturated compounds while suppressing less polar, N-containing, and some high-molecular weight species, rendering portions of the DOM pool effectively undetectable [14,52]. Differences in ionization efficiency among compounds further limit quantitative interpretation, as peak intensity does not necessarily reflect concentration. In addition, the solid-phase extraction technique used herein to isolate and C-normalize DOM can selectively retain certain fractions of DOM while excluding others, thereby influencing the apparent molecular composition [53]. Accordingly, the FT-ICR MS results reflect shifts within the ionizable and extractable fraction of DOM rather than the complete DOM pool. These methodological constraints may influence the apparent extent and specificity of PVPP binding by underrepresenting compounds that are not efficiently extracted or ionized. Nevertheless, the clear loss of aromatic molecular formulae and associated DOM compositional shifts highlight what we would expect from PVPP in such experiments.

## Conclusions

PVPP provides a robust experimental tool for isolating phenolic effects on microbial activity and carbon turnover. However, even under optimized conditions, this method was not successful at removing all traces of phenolic compounds, leaving behind ~10% of their initial abundance. Yet this approach co-removes a substantial fraction of other DOM constituents in addition to phenolic moieties (i.e., condensed aromatic molecular formulae), which should be considered when interpreting experimental results. Future work applying this method to natural porewaters across peatland types will further establish its utility and refine its limitations and may help explain variability in PVPP effectiveness across sites with different phenolic molecular compositions. Incorporating PVPP treatments and molecular-level DOM characterization alongside complementary incubation-based approaches [4] may further strengthen mechanistic tests of phenolic controls on peatland carbon cycling.
